# Inhibitors Incorporating Zinc-Binding Groups Target the GlcNAc-PI de-*N*-acetylase in *Trypanosoma brucei*, the Causative Agent of African Sleeping Sickness

**DOI:** 10.1111/j.1747-0285.2011.01300.x

**Published:** 2012-03

**Authors:** Nuha Z Abdelwahab, Arthur T Crossman, Lauren Sullivan, Michael A J Ferguson, Michael D Urbaniak

**Affiliations:** Division of Biological Chemistry and Drug Discovery, College of Life Sciences, University of DundeeDundee DD1 5EH, UK

**Keywords:** carbohydrates, glycosylphosphatidylinositol, lipid, mechanism-based drug design, metalloenzymes, *Trypanosoma brucei*

## Abstract

Disruption of glycosylphosphatidylinositol biosynthesis is genetically and chemically validated as a drug target against the protozoan parasite *Trypanosoma brucei*, the causative agent of African sleeping sickness. The *N*-acetylglucosamine-phosphatidylinositol de-*N*-acetylase (deNAc) is a zinc metalloenzyme responsible for the second step of glycosylphosphatidylinositol biosynthesis. We recently reported the synthesis of eight deoxy-2-*C*-branched monosaccharides containing carboxylic acid, hydroxamic acid, or *N*-hydroxyurea substituents at the C2 position that may act as zinc-binding groups. Here, we describe the synthesis of a glucocyclitol-phospholipid incorporating a hydroxamic acid moiety and report the biochemical evaluation of the monosaccharides and the glucocyclitol-phospholipid as inhibitors of the trypanosome deNAc in the cell-free system and against recombinant enzyme. Monosaccharides with carboxylic acid or hydroxamic acid substituents were found to be the inhibitors of the trypanosome deNAc with IC_50_ values 0.1–1.5 mm, and the glucocyclitol-phospholipid was found to be a dual inhibitor of the deNAc and the α1-4-mannose transferase with an apparent IC_50_ = 19 ± 0.5 μm.

Glycosylphosphatidylinositol (GPI)-anchored proteins are abundant in the protozoan parasite *Trypanosoma brucei*, the causative agent of African sleeping sickness in humans and the related disease Nagana in cattle (1), and disruption of GPI biosynthesis has been genetically (2–4) and chemically (5) validated as a drug target. The clinically relevant bloodstream form of *T. brucei* expresses approximately 5 × 10^6^ GPI-anchored variant surface glycoprotein homodimers that form a dense surface coat, protecting the parasite from the complement pathway of the host and undergoing antigenic variation to evade specific immune responses (6,7). African sleeping sickness is invariably fatal if untreated and kills about 50 000 people each year (8). Current drugs are toxic, expensive, and difficult to administer, leaving an urgent need for new therapeutic agents.

The structure, biosynthesis, and function of GPIs and related molecules have been extensively reviewed (1,9–11). The basic conserved GPI core of NH_2_CH_2_CH_2_PO_4_H-6Manα1-2Manα1-6Manα1-4GlcNα1-6-d-*myo-*inositol-1-HPO_4_-lipid, where the lipid can be diacylglycerol, alkylacylglycerol, or ceramide, is often further decorated with additional ethanolamine phosphate and/or carbohydrate groups in a species- and tissue-specific manner. Biosynthesis of GPI, which occurs in the endoplasmic reticulum, is initiated by the transfer of *N*-acetylglucosamine (GlcNAc) from UDP-GlcNAc to phosphatidylinositol (PI) to generate *N*-acetylglucosamine-phosphatidylinositol (GlcNAc-PI **1**, Figure 1), which is de-*N-*acetylated by the enzyme GlcNAc-PI de-*N*-acetylase (EC3.5.1.89) to give GlcN-PI **2** (12). This de-*N*-acetylation is a prerequisite for the subsequent mannosylation of GlcN-PI that leads to mature GPI anchor precursors (13). From GlcN-PI onwards, there are significant differences in the GPI biosynthetic pathways of *T. brucei* and mammalian cells (14–17).

**Figure 1:**
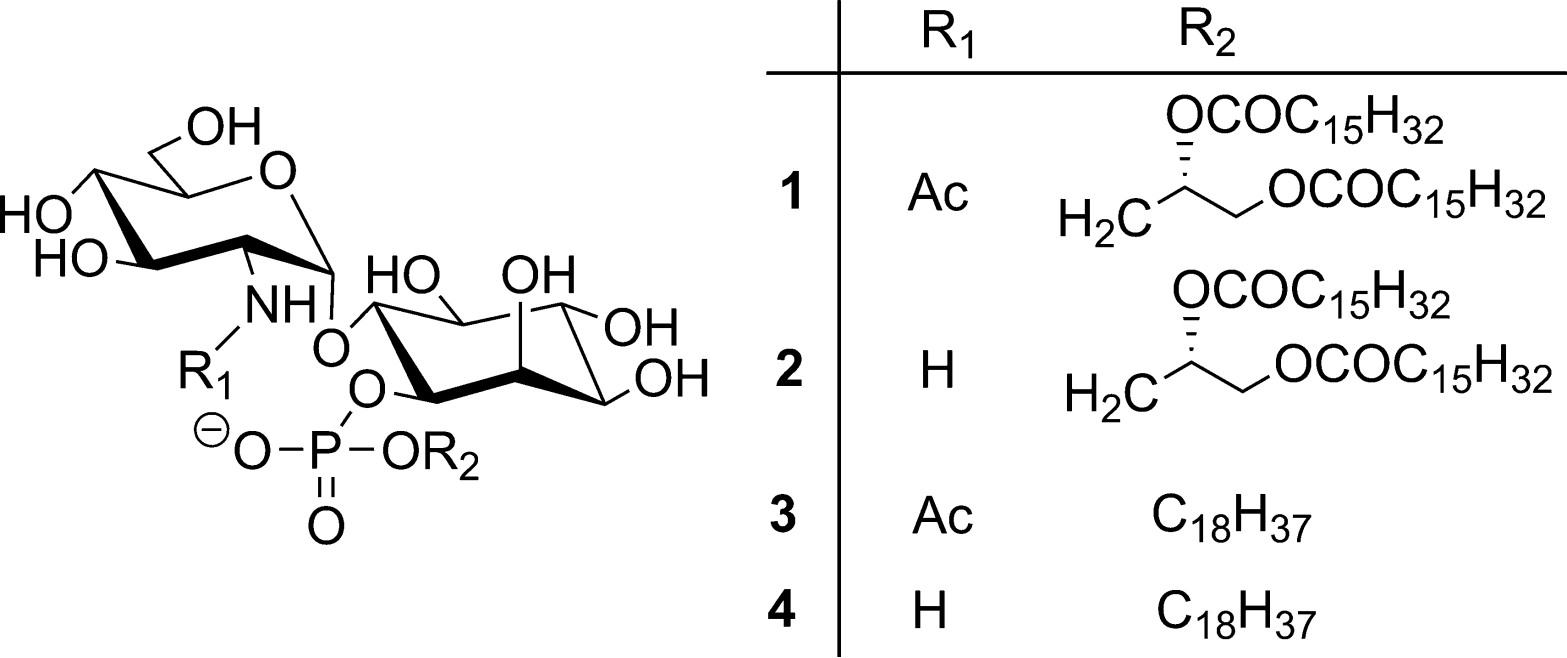
The GlcNAc-PI de-*N*-acetylase substrates and products used in this study.

No high-resolution structural data exist for any of the enzymes of the GPI biosynthetic pathway, and given that the enzymes contain between one and 13 predicted transmembrane domains and/or are components of multi-protein complexes, such structural data may prove difficult to obtain. Instead, the substrate specificity of the enzymes of the *T. brucei* and HeLa GPI biosynthetic pathways has been examined *in vitro* using a substrate analog approach (5,13–16,18–21). The *T. brucei* enzymes have less stringent substrate recognition than those of the mammalian pathway, enabling substrate-based species-specific inhibitors to be designed (16,18).

The *T. brucei* GlcNAc-PI de-*N*-acetylase (deNAc) has been genetically validated as a drug target through the generation of a conditional null mutant (3) and has been shown to be a zinc metalloenzyme (22). Owing to their role in the progression of various human diseases, zinc metalloenzymes have gained much interest as potential drug targets (23,24). Synthetic inhibitors of zinc metalloenzymes typically consist of a backbone and a zinc-binding group (ZBG). The backbone is typically a drug-like structure that interacts with the protein through non-covalent interactions and contributes to both the affinity and selectivity of the inhibitor for its target. The ZBG coordinates to the zinc divalent cation and primarily contributes to the binding affinity of the inhibitor–metalloenzyme complex.

We have postulated that ZBGs could act as inhibitors of the *T. brucei* GlcNAc-PI de-*N*-acetylase (22). As part of our efforts to test this hypothesis, we have previously reported the synthesis of a GlcNAc-PI analog incorporating an *N*-hydroxyurea zinc-binding moiety (1-d-6-*O*-[2-(*N*-hydroxyaminocarbonyl)amino-2-deoxy-α-d-glucopyranosyl]-*myo*-inositol 1-(*n*-octadecyl phosphate) (25). Unfortunately, this compound proved to be unstable under the conditions employed in the activity assay and was therefore judged to be unsuitable for further study. We recently reported the synthesis of eight deoxy-2-*C*-branched monosaccharides incorporating ZBGs, Figure 2 (26). Here, we describe the synthesis of a glucocyclitol-phospholipid incorporating a ZBG and report the biological evaluation of the monosaccharides and the glucocyclitol-phospholipid as inhibitors of the trypanosome GlcNAc-PI de-*N*-acetylase.

**Figure 2:**
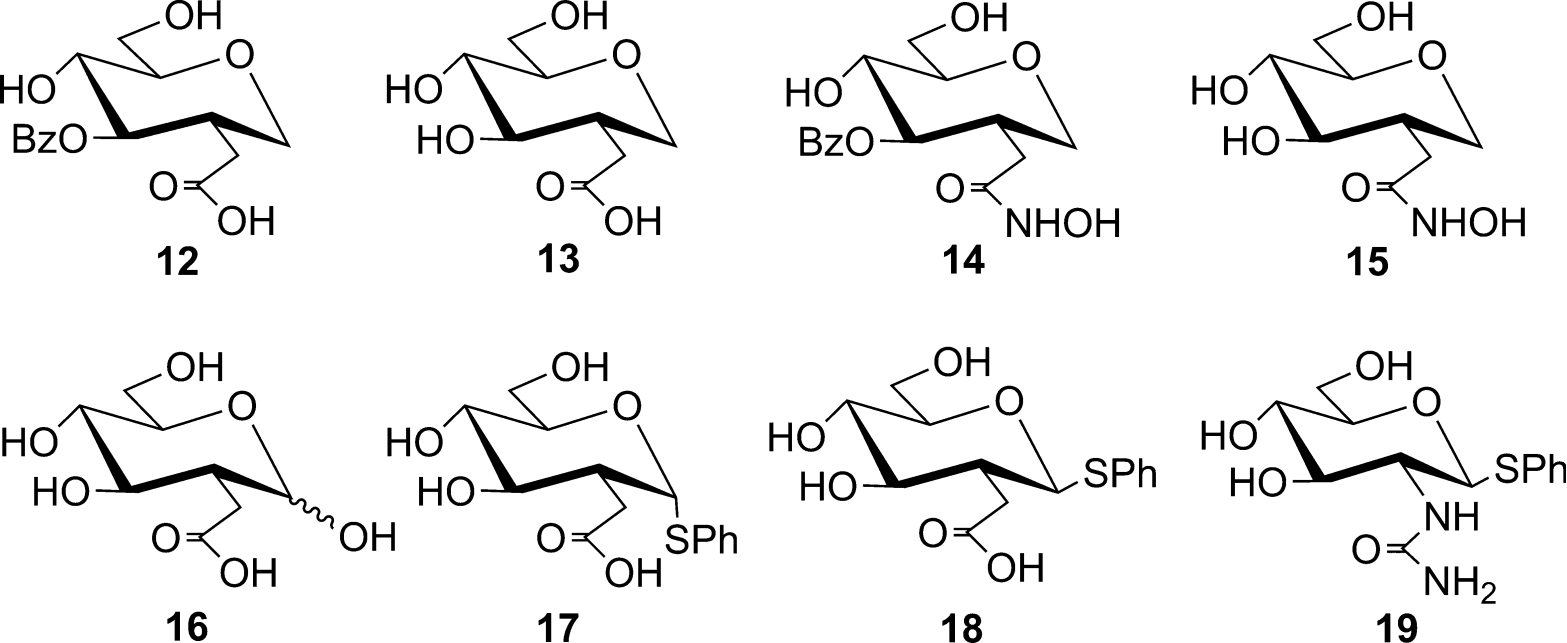
Structures of the deoxy-2-*C*-branched monosaccharides.

## Materials and Methods

### General methods

^1^H, ^13^C, ^31^P NMR spectra were recorded on a Bruker AVANCE spectrometer using deuteriochloroform as a solvent and tetramethylsilane as the internal standard, unless otherwise indicated. All coupling constants (*J*) are given in Hz. High-resolution electrospray ionization mass spectra (HRESIMS) and liquid chromatography mass spectra were recorded with a Bruker microTof spectrometer. Melting points were determined on a Reichert hot-plate apparatus and are uncorrected. Optical rotations were measured with a Perkin-Elmer 343 polarimeter. Thin-layer chromatography (TLC) was performed on Kieselgel 60 F_254_ (Merck, Nottingham, UK) with various solvent systems as developers, followed by detection under UV light or by charring using either sulfuric acid/water/ethanol (15:85:5), phosphomolybdic acid, orcinol, or ninhydrin spray reagents. Flash column chromatography (FCC) was performed on Kieselgel 60 (0.040−0.063 mm) (Merck, Notttingham, UK). Radial-band chromatography (RBC) was performed using a Chromatotron (model 7924T; TC Research, Norwich, UK) with silica gel F_254_ TLC standard grade as the adsorbent. All reactions were carried out in commercially available dry solvents, unless otherwise stated.

### Substrates and deoxy-2-C-branched monosaccharides

The synthesis of d-GlcN-α(1–6)-d-*myo*-inositol-1-octadecyl phosphate (GlcN-I*P*C_18_, **3**) (27) and the monosaccharides **12**–**19** has been described previously (26). The corresponding *N*-acetyl derivate GlcNAc-I*P*C_18_**4** was prepared by treatment with acetic anhydride (14). The concentration of stock solutions of **3** and **4** was determined by measurement of the inositol content by selected ion-monitoring GC-MS (28).

### Phenyl 3,4,6-tri-O-acetyl-2-C-(carboxymethyl N-benzyloxyamide)-2-deoxy-1-thio-d-glucopyranoside (**6**)

EDAC (58.7 mg, 0.31 mmol) and TEA (40 μL, 0.30 mmol) were added successively to a solution of the carboxylic acid **5** (26) (90 mg, 0.21 mmol) and *O*-benzylhydroxylamine hydrochloride (39 mg, 0.25 mmol) in CH_2_Cl_2_ (3 mL) under argon at room temperature. After 2 h, TLC showed the complete disappearance of the starting carboxylic acid whereby CH_2_Cl_2_ (10 mL) was added, and the resulting solution was washed with water (5 mL), brine (5 mL), dried with Na_2_SO_4_, filtered, and concentrated under reduced pressure. The crude product was purified by RBC (5:1→1:1 hexane−EtOAc) to give the benzyloxyamide **6** (89 mg, 80%): R_f_ 0.27 (1:1 hexane−EtOAc); ^1^H NMR (CDCl_3_, 500 MHz): δ 9.60, 9.40 (2 × s, α & β NH), 7.50−7.20 (m, 4 × Ph), 5.70 (d, *J*_1,2_ 3.8 Hz, H-1α), 5.26 (t, *J*_2,3_ = *J*_3,4_ 9.9 Hz, H-3β), 5.15 (t, *J*_2,3_ = *J*_3,4_ 9.9 Hz, H-3α), 5.02 (t, *J*_4,5_ 10.1 Hz, H-4α), 4.95−4.77 (m, H-*1*β, H-4β, C*H*_2_Ph), 4.57−4.52 (m, H-5α), 4.26–4.17 (m, H-6aα, H-6a,bβ), 4.10–4.00 (m, H-6bα, C*H*_2_Ph), 3.70−3.65 (m, H-5β), 3.03−2.97 (m, H-2α), 2.40−2. 32 (m, H-2β, H-7a,bβ), 2.29−2.20 (m, H-7a,bα), 2.13, 2.03, 1.99, 1.98,1.97, 1.96 (6 × s, 6 × CH_3_CO); ^13^C NMR (CDCl_3_, 125 MHz): δ 174.2, 173.7, 170.8, 170.7, 170.5, 169.8, 169.8, 168.0 (8 × C=O), 135.5−127.6 (C-Ph), 87.5 (C-1α), 86.4 (C-1β), 79.3, 79.1 (*C*H_2_Ph), 75.2 (C-5β), 74.1 (C-3β), 72.2 (C-3α), 69.9 (C-4α), 69.7 (C-4β), 68.7 (C-5α), 62.6 (C-6β), 62.4 (C-6α), 41.8 (C-2β), 41.5 (C-2α), 32.6 (C-7α), 31.5 (C-7β), 21.0, 20.9, 20.8, 20.7, 20.7, 20.6 (6 × s, 6 × *C*H_3_CO). HRESIMS: Calcd for [C_27_H_31_NO_9_S + H]^+^: 546.1792. Found *m/z*: 546.1805.

### Phenyl 3,4,6-tri-O-acetyl-2-C-(carboxymethyl N-tert-butyloxycarbonyl-N-benzyloxy-amide)-2-deoxy-d-glucopyranoside (**7**)

Di-*tert*-butyl dicarbonate (0.30 g, 1.4 mmol) was added to a stirred solution of the benzyloxyamide **6** (0.15 g, 0.28 mmol) and 4-(dimethylamino) pyridine (DMAP) (3.4 mg, 0.03 mmol) in THF (8 mL). Stirring was continued at room temperature for 3.5 h, and then the reaction mixture was concentrated under reduced pressure. The crude product was purified by RBC (6:1→2:1 hexane−EtOAc) to yield an α:β (4:1) anomeric mixture of the *N*-Boc derivative **7** (173 mg, 96%): R_f_ 0.26 (2:1 hexane−EtOAc); ^1^H NMR (CDCl_3_, 500 MHz): δ 7.52−7.24 (m, 4 × Ph), 5.91 (d, *J*_1,2_ 5.0 Hz, H-1α), 5.41 (dd, *J*_2,3_ 10.6, *J*_3,4_ 7.2 Hz, H-3β), 5.27 (d, *J*_1,2_ 10.8 Hz, H-1β), 5.21 (dd, *J*_2,3_ 11.4, *J*_3,4_ 9.1 Hz, H-3α), 5.04 (dd, *J*_4,5_ 10.2 Hz, H-4α), 4.98 (dd, *J*_4,5_ 10.1 Hz, H-4β), 4.91−4.84 (m, 2 × C*H*_2_Ph), 4.59−4.55 (m, H-5α), 4.31 (dd, *J*_5,6a_ 5.1, *J*_6a,6b_ 12.3 Hz, H-6aα), 4.26 (dd, *J*_5,6a_ 5.5, *J*_6a,6b_ 12.2 Hz, H-6aβ), 4.16 (dd, *J*_5,6b_ 2.2 Hz, H-6bβ) 4.05 (dd, *J*_5,6b_ 2.1 Hz, H-6bα), 3.80−3.76 (m, H-5β), 3.17 (dd, *J*_2,7a_ 4.3, *J*_7a,7b_ 17.1 Hz, H-7aβ), 3.08−3.03 (m, H-7aα, H-7bβ), 3.01−2.96 (m, H-2α), 2.87 (dd, *J*_2,7b_ 4.1, *J*_7a,7b_ 17.1 Hz, H-7bα), 2.41−2.35 (m, H-2β), 2.08, 2.05, 2.04, 2.03, 2.01, 2.00 (6 × s, 6 × CH_3_CO), 1.54, 1.53 (2 × s, (C*H*_3_)_3_C); ^13^C NMR (CDCl_3_, 125 MHz): δ 170.7, 170.5, 170.4, 169.9, 169.8, 169.1, 169.0, 169.0 (8 × C=O), 151.2, 150.5 [(CH_3_)_3_CO*C*=O], 134.4−127.6 (C-Ph), 87.5 (C-1α), 86.2 (C-1 β), 78.1, 77.8 (*C*H_2_Ph), 75.5 (C-5β), 73.6 (C-3β), 72.0 (C-3α), 69.9 (C-4α), 69.6 (C-4β), 68.6 (C-5α), 62.7 (C-6β), 62.4 (C-6α), 42.4 (C-2β), 41.5 (C-2α), 36.6 (C-7α), 34.7 (C-7β), 28.0, 27.4 [2 × (*C*H_3_)_3_C], 20.8, 20.7, 20.7 (3 × s, 6 × *C*H_3_CO). HRESIMS: Calcd for [C_32_H_39_NO_11_S + Na]^+^: 668.2086. Found *m/z*: 668.2098.

### 1R,2R-1-O-[3,4,6-Tri-O-acetyl-2-C-(carboxymethyl N-tert-butyloxycarbonyl-N-benzyl-oxyamide)-2-deoxy-α-d-glucopyranosyl]-cyclohexanediol (**8**) and the β-anomer (**9**)

A freshly prepared 0.4 m benzenesulfenyl chloride solution (29) (0.68 mL, 0.27 mmol) was added dropwise to a solution of silver triflate (AgOTf) (83 mg, 0.32 mmol) in CH_2_Cl_2_ (1.5 mL) under argon at −78 °C. While at −78 °C, the mixture was stirred for a further 5 min and then activated powdered 4 Å molecular sieves (20 mg) were added; followed by the dropwise addition of a solution of the *N*-Boc protected hydroxamate **7** (60 mg, 0.93 mmol) and 2,6-di-*tert*-butyl-4-methyl pyridine (DTBMP) (67 mg, 0.33 mmol) in CH_2_Cl_2_ (0.5 mL). After a further 5 min at −78 °C, a solution of 1R,2R-*trans*-cyclohexanediol (32 mg, 0.28 mmol) in THF−CH_2_Cl_2_ (1:1 0.5 mL) was added dropwise. The reaction mixture was continuously stirred under argon at −78 °C for 2 h and then allowed to gradually attain room temperature over a 30-min period. Afterward, the mixture was filtered through a pad of Celite and concentrated under reduced pressure. The crude material was purified by RBC (1:1→1:3 cyclohexane−Et_2_O) to give first the β-anomer **9** (36 mg, 60%): mp 111−115 °C; R_f_ 0.22 (1:3 cyclohexane−Et_2_O); 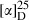-11.0 (*c* 1.0, CHCl_3_); ^1^H NMR (CDCl_3_, 500 MHz): δ 7.48−7.36 (m, 5H, Ph), 5.33 (dd, 1H, *J*_2,3_ 11.1, *J*_3,4_ 9.2 Hz, H-3), 5.03 (d, 1H, *J*_1,2_ 8.6 Hz H-1), 5.00−4.86 (m, 3H, H-4 and C*H*_2_Ph), 4.24 (dd, 1H, *J*_5,6a_ 5.5, *J*_6a,6b_ 12.2 Hz, H-6a), 4.16 (dd, 1H, *J*_5,6b_ 2.4 Hz, H-6b), 3.80 (ddd, 1H, *J*_4,5_ 10.1 Hz, H-5), 3.41−3.36 (m, 1H, H-1′), 3.34−3.29 (m, 1H, H-2′), 3.13 (dd, 1H, *J*_2,7a_ 4.8, *J*_7a,7b_ 17.8 Hz, H-7a), 2.85 (dd, 1H, *J*_2,7b_ 4.3 Hz, H-7b), 2.34−2.27 (m, 1H, H-2), 2.09, 2.02, 2.01 (3 × s, 9 H, 3 × CH_3_CO), 2.06–2.03 (m, 1H, H-6′a), 1.89−1.84 (m, 1H, H-3′a), 1.66 (br, 2H, H-4′a, H-5′a), 1.53 (s, 9H, (CH_3_)_3_C), 1.30−1.22 (m, 2H, H-3′b, H-4′b), 1.20−1.16 (m, 2H, H-5′b, H-6′b); ^13^C NMR (CDCl_3_, 125 MHz): δ 170.8, 170.6, 169.8, 169.2 (4 × C=O), 151.2 [(CH_3_)_3_COC=O], 134.3−128.5 (C-Ph), 102.0 (C-1), 87.1 (C-2′), 77.9 (CH_2_Ph), 73.2 (C-1′), 72.5 (C-3), 71.7 (C-5), 69.6 (C-4), 62.2 (C-6), 43.6 (C-2), 32.8 (C-7), 32.2 (C-6′), 30.9 (C-3′), 28.0 [(CH_3_)_3_C], 24.3, 23.7 (C-4′, C-5′), 20.8, 20.7, 20.7 (3 × CH_3_CO). HRESIMS: Calcd for [C_32_H_45_NO_13_ + Na]^+^: 674. 2783. Found *m/z*: 674.2761. Then the α-anomer **8** (7.9 mg, 13%): mp 99−103 °C; R_f_ 0.18 (1:3 cyclohexane − Et_2_O); 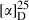 + 36.0 (*c* 1.0, CHCl_3_); ^1^H NMR (CDCl_3_, 500 MHz): δ 7.48−7.35 (m, 5H, Ph), 5.89 (d, 1H, *J*_1,2_ 5.0 Hz, H-1), 5.08−5.01 (m, 4H, H-3, H-4 and C*H*_2_Ph), 4.38 (dd, 1H, *J*_5,6a_ 4.8, *J*_6a,6b_ 12.4 Hz, H-6a), 4.16 (dd, 1H, *J*_5,6b_ 2.5 Hz, H-6b), 4.08−4.05 (m, 1H, H-5), 3.44−3.39 (m, 1H, H-1′), 3.20−3.15 (m, 1H, H-2′), 2.81−2.67 (m, 3H, H-2, H-7a,b), 2.10, 2.08, 2.05 (3 × s, 9H, 3 × CH_3_CO), 2.09 (br, 1H, H-3′a), 2.04–2.00 (m, 1H, H-6′a), 1.75−1.69 (m, 2H, H-4′a, H-5′a), 1.43 (s, 9H, (CH_3_)_3_C), 1.29−1.24 (m, 2H, H-5′b, H-6′b), 1.24−1.11 (m, 2H, H-3′b, H-4′b); ^13^C NMR (CDCl_3_, 125 MHz): δ 171.8, 170.5, 170.0, 169.5 (4 × C=O), 152.5 [(CH_3_)_3_COC=O], 134.0−129.0 (C-Ph), 100.3 (C-1), 80.4 (C-2′), 78.0 (CH_2_Ph), 73.4 (C-1′), 71.5 (C-3), 70.0 (C-5), 67.3 (C-4), 61.8 (C-6), 39.9 (C-2), 34.3 (C-7), 32.2 (C-6′), 30.3 [(CH_3_)_3_C], 29.7 (C-3′), 24.4, 24.0 (C-4′, C-5′), 20.7, 20.7, 20.6 (3 × CH_3_CO). HRESIMS: Calcd for [C_32_H_45_NO_13_ + Na]^+^: 674. 2783. Found *m/z*: 674.2761.

### Triethylammonium 1R,2R-1-O-[3,4,6-tri-O-acetyl-C-(carboxymethyl N-tert-butyloxy-carbonyl-N-benzyloxyamide)-2-deoxy-β-d-glucopyranosyl]-cyclohexanediol 2-(n-octa-decyl phosphate) (**10**)

Trimethylacetyl chloride (66 μL, 0.54 mmol) was added to a stirred mixture of the pseudo-disaccharide **9** (56 mg, 0.086 mmol) and the *n*-octadecyl hydrogenphosphonate (27) (75 mg, 0.17 mmol) in pyridine (5 mL) under argon at room temperature. The reaction mixture was stirred for 2 h, and then a solution of iodine (87 mg, 0.344 mmol) and water (0.1 mL) in pyridine (9.5 mL) was subsequently added. Stirring of the mixture was continued for 45 min, after which it was diluted with dichloromethane (40 mL), washed with 5% sodium hydrogen sulfite (20 mL), water (15 mL), 1.0 m triethylammonium hydrogen carbonate (TEAB) buffer solution (3 × 20 mL), dried (MgSO_4_), and concentrated under reduced pressure. The residue was purified by column chromatography (5:1 CHCl_3_− MeOH) to give the TEA salt **10** (54 mg, 58%): R_f_ 0.28 (5:1 CHCl_3_− MeOH); 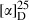-8.4 (*c* 1.0, CHCl_3_); ^1^H NMR (CDCl_3_, 500 MHz): δ 12.91 (s, 1H, N*H*(CH_2_CH_3_)_3_), 7.47−7.35 (m, 5H, Ph), 5.35 (dd, 1H, *J*_2,3_ 11.1, *J*_3,4_ 9.2 Hz, H-3), 5.06 (d, 1H, *J*_1,2_ 8.6 Hz, H-1), 5.02 (dd, 1H, *J*_4,5_ 9.9 Hz, H-4), 4.95 and 4.86 (ABq, 2H, *J* 9.2 Hz, C*H*_2_Ph), 4.31−4.28 (m, 2H, H-2′, H-6a), 4.09 (dd, 1H, *J*_5,6b_ 2.6, *J*_6a,6b_ 12.1 Hz, H-6b), 3.95−3.85 (m, 2H, OCH_2_), 3.82−3.78 (m, 1H, H-1′), 3.74 (ddd, 1H, *J*_5,6a_ 4.0 Hz, H-5), 3.10 (dd, 1H, *J*_2,7a_ 4.4, *J*_7a,7b_ 18.0 Hz, H-7a), 3.07−3.02 (m, 6H, HN(C*H*_2_CH_3_)_3_), 2.89 (dd, 1H, *J*_2,7b_ 4.4 Hz, H-7b), 2.31−2.25 (m, 1H, H-2), 2.09, 2.05, 2.00 (3 × s, 9H, 3 × CH_3_CO), 1.99−1.93 (m, 1H, H-3′a), 1.91−1.85 (m, 1H, H-6′a), 1.72−1.58 (m, 4H, H-3′b, H-4′a, OCH_2_C*H*_2_), 1.52 (s, 9H, (CH_3_)_3_C), 1.50−1.45 (m, 1H, H-5′a), 1.44−1.38 (m, 1H, H-6′b), 1.32−1.24 (m, H-4′b, H-5′b, 3 × CH_2_C*H*_3_, (CH_2_)_15_), 0.92−0.86 (m, 3H, CH_2_C*H*_3_); ^13^C NMR (CDCl_3_, 125 MHz): δ 170.9, 170.6, 169.8, 169.4 (4 × C=O), 151.0 [(CH_3_)_3_COC=O], 134.4−128.5 (C-Ph), 99.7 (C-1), 77.8 (CH_2_Ph), 77.5 (C-1′), 74.4 (br, C-2′), 72.8 (C-3), 71.5 (C-5), 69.7 (C-4), 66.1 (br, OCH_2_), 62.2 (C-6), 45.2 (CH_2_CH_3_), 43.6 (C-2), 32.7 (C-7), 31.7 (OCH_2_CH_2_), 30.7 (C-6′), 30.7 (C-3′), 29.7 (CH_2_CH_3_), 29.5, 29.4 (CH_2_), 28.0 [(CH_3_)_3_C], 27.0, 25.8, 25.5, 22.7, 21.6 (CH_2_), 20.8, 20.7 (CH_3_CO), 14.2, 8.5 (CH_3_); ^31^P NMR (CDCl_3_, 202 MHz): δ_P_−0.86 (with ^1^H heteronuclear decoupling). HRESIMS: Calcd for [C_50_H_81_NO_16_P-NEt_3_-H]^−^: 982.5298. Found *m/z*: 982.5380.

### Triethylammonium 1R,2R-1-O-[2-C-(carboxymethyl N-hydroxyamide)-2-deoxy-β-d-glucopyranosyl]-cyclohexanediol 2-(n-octadecyl phosphate) (**11**)

A 0.03 m methanolic sodium methoxide solution (0.36 mL, 0.011 mmol) was added to a solution of the TEA salt **10** (15 mg, 0.014 mmol) in methanol (1 mL) under argon at room temperature, and the reaction was stirred for 6 h. Afterward, the reaction mixture was neutralized with Amberlite IR-120 (H^+^) ion-exchange resin, filtered, and the filtrate was concentrated under reduced pressure and then co-evaporated with water (5 × 5 mL). The residue was purified by FCC (5:1→3:1 CHCl_3_− MeOH) to give the hydroxamic acid TEA salt **11** (6 mg, 56%): R_f_ 0.21 (3:1 CHCl_3_− MeOH); 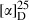
−2.0 (*c* 0.5, CHCl_3_); ^1^H NMR (CDCl_3_, 500 MHz): δ 12.30 (s, 1H, N*H*(CH_2_CH_3_)_3_), 8.00 (s, 1H, NH), 4.42 (d, 1H, *J*_1,2_ 8.6 Hz, H-1), 4.22 (br, 1H, H-2′), 3.88−3.79 (m, 4H, H-6a, H-6b and OCH_2_), 3.70 (br, 1H, H-1′), 3.47–3.41 (m, 1H, H-4), 3.37 (dd, 1H, *J*_2,3_ 9.2, *J*_3,4_ 8.0 Hz, H-3), 3.27 (br, 1H, H-5), 3.11−3.02 (m, 6H, (C*H*_2_CH_3_)_3_), 2.64 (dd, 1H, *J*_2,7a_ 4.1, *J*_7a,7b_ 15.2 Hz, H-7a), 2.45 (dd, 1H, *J*_2,7b_ 6.5 Hz, H-7b), 2.12−2.05 (m, 1H, H-2), 1.64−1.48 (m, 3H, H-3′a and OCH_2_C*H*_2_), 1.40−1.21 (m, 46H, H-3′b, H-4′a,b, H-5′a,b, H-6′a,b, (CH_2_C*H*_3_)_3_, (CH_2_)_15_), 0.88 (t, 3H, *J* 6.7 Hz, CH_2_C*H*_3_); ^13^C NMR (CDCl_3_, 125 MHz): δ 169.5 (CONOH), 100.6 (C-1), 76.9 (br, C-1′), 75.6 (C-5), 74.0 (C-3), 73.7 (br, C-2′), 70.5 (C-4), 64.7 (br, OCH_2_), 60.6 (C-6), 44.4 [(*C*H_2_CH_3_)_3_], 43.8 (C-2), 31.4 (C-7), 30.9−20.8 (CH_2_), 13.1 (CH_3_), 7.5 [(CH_2_*C*H_3_)_3_]; ^31^P NMR (CDCl_3_, 202 MHz): δ_P_−0.70 (with ^1^H heteronuclear decoupling). HRESIMS: Calcd for [C_32_H_61_NO_11_P-NEt_3_-H]^−^: 666.3988. Found *m/z*: 666.4045.

### Trypanosome cell-free system inhibition assays

Bloodstream form *T. brucei* (variant MITat1.4) were isolated and membranes (cell-free system) prepared as described previously and stored at −80 °C (30). Trypanosome membranes (2 × 10^7^ cell equivalents per assay) were washed twice in incorporation buffer (50 mm NaHEPES pH 7.4, 25 mm KCl, 0.1 mm Tos-LysCH_2_Cl and 1 μg/mL leupeptin) and resuspended in incorporation buffer (40 μL per assay) supplemented with 5 mm MnCl_2_, 5 mm MgCl_2_, 5 mm*N*-ethylmaleimide, 0.15%*n-*octyl β-d-glucopyranoside, 1.25 μg/mL tunicamycin and GDP-[^3^H]Man (0.5 μCi per assay), and briefly sonicated. The cell-free system was incubated with or without inhibitor for 5 min at 30 °C, transferred to tubes containing 400 pMol dry GlcNAc-I*P*C_18_ or GlcN-C_18_, sonicated briefly and incubated at 30 °C for 30 min. Glycolipid products were recovered by extraction into a chloroform/methanol/water mixture (10:10:3), evaporated to dryness, partitioned between butan-1-ol and water, and analyzed by high-performance thin-layer chromatography (hptlc).

### High-performance thin-layer chromatography

Glycolipid standards and samples were applied to 10 cm aluminum-backed silica gel 60 and developed with chloroform/methanol/13 m ammonia/1 m ammonium acetate/water (180/140/9/9/23, v/v). Dried hptlc plates were analyzed by a radiometric scanner (Bioscan AR2000, Washington, DC, USA) and/or sprayed with En^3^Hance™ (PerkinElmer, Boston, MA, USA) and radiolabeled components visualized by fluorography at −80 °C using Kodak XAR-5 film (Sigma, St. Louis, MO, USA) with an intensifying screen.

### Cloning of T. brucei GlcNAc-PI de-N-acetylase

The DNA-encoding residues 24–252 of *T. brucei* GlcNAc-PI de-*N*-acetylase (Tb11.01.3900) was amplified by PCR from genomic *Trypanosoma brucei brucei* DNA (variant MITat1.4) using a 5′ primer (5′-ttatact*ggatcc*atggataaggttttagatgcatcttgcgtaagt-3′) that incorporated an *BamH*I site (italics) and a six residue N-terminal sequence (MDKVLD, underlined) and a 3′- primer (5′-tataat*gcggccgc*tcatgcgacccccaat-3′) that incorporated a *Not*I site (italics). The PCR fragment was subcloned into pGEX-6P-1 (Invitrogen) with *BamH*I and *Not*I to give the plasmid pGEX-*TbGPI12*, which appended a GST tag to the N-terminus of the ORF.

### Expression of recombinant T. brucei GlcNAc-PI de-N-acetylase

*Escherichia coli* BL21 (DE3) transformed with pETB-*TbGPI12* were grown in Luria-Bertani medium with 50 μg/mL carbenicillin at 37 °C until A_600_∼ 0.5, induced with 250 μm isopropyl β-d-thiogalactoside, and cultured for a further 16 h at 21 °C. Cells were harvested by centrifugation at 4500 × *g* for 20 min at 4 °C, resuspended in 10 mL buffer A (50 mm Tris–HCl pH 8.0, 200 mm NaCl, 0.06%*n*-octyl-β-d-glucopyranoside, 10% glycerol v/v) per liter, and incubated with lysozyme (1 mg/mL) on ice for 20 min. Cells were lysed at 30 000 psi (OneShot; Constant Cell Disruption Systems, Daventry, UK), and the lysate clarified by centrifugation at 30 000 × *g* for 30 min at 4 °C. The supernatant was filtered through a 0.4 μm membrane, mixed with glutathione-sepharose beads (GE Healthcare, Chafont St. Giles, UK) for 2 h at 4 °C, washed with buffer A, eluted with 20 mm glutathione in buffer A. Pooled fractions were concentrated and washed in buffer A in a 10 000 molecular weight cut-off spin-concentration device (Satorius, Aubagne, France) repeatedly to remove glutathione. The concentration of GST-TbGPI12 was determined by absorbance at 280 nm using a calculated ε = 7.464 × 10^4^/cm/m.

### Tryptic peptide mass fingerprinting of GST-TbGPI12

The protein was reductively alkylated prior to SDS-PAGE and staining with Sypro Orange, the band excised and digested in 0.1% n-octylglucoside and 20 mm NaHCO_3_ with 12.5 μg/mL trypsin before analysis by MALDI-TOF MS and MS-MS. The protein was confirmed as *T. brucei* GlcNAc-PI de-*N*-acetylase with a Mascot score of 1162, with 87% sequence coverage.

### ES-MS/MS de-N-acetylase assay

The recombinant protein GST-TbGPI12 (500 ng/μL) was incubated in incorporation buffer (50 μL) with or without inhibitor for 5 min at RT. The solution was transferred to tubes containing dry GlcN-I*P*C_18_**3** (0.5 nmol), briefly vortexed, sonicated for 5 seconds, and incubated at 37 °C for 30 min. The mixture was diluted with 5% propan-1-ol (1 mL), the glycolipids were bound to C8 resin (100 mg Isolute cartridge), washed (5% propan-1-ol, 5 mm NH_4_OAc), eluted (60% propan-1-ol, 5 mm NH_4_OAc), and the products analyzed directly by electrospray tandem mass spectrometry (Micromass Quattro Ultima, Manchester, UK) in precursor ion scanning mode (precursor of *m/z* 223). The *m/z* 223 fragment ion, [inositol-1,2-cyclic phosphate – H_2_O], is common to both the GlcNAc-I*P*C_18_ substrate **3** and the GlcN-I*P*C_18_ product **4**. The ratio of the integrals for the *m/z* 714 [GlcNAc-I*P*C_18_– H]- and *m/z* 672 [GlcN-I*P*C_18_– H]- precursor ions were used to calculate the percentage of substrate conversion to product in a given sample. Inhibitor IC_50_ values were calculated using a four-parameter fit of eight-point potency curves derived from three independent experiments, and are quoted with standard deviation.

## Results and Discussion

### Synthesis of the glucocyclitol-phospholipid

**Figure 3:**
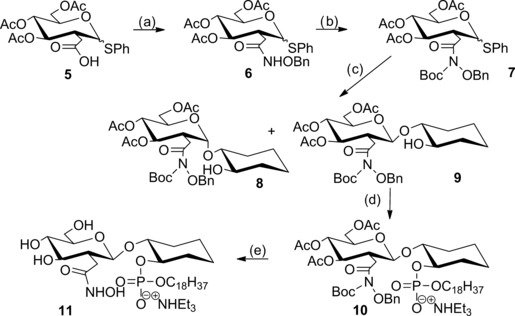
Synthesis of compound **11**. Experimental conditions: (a) BnONH_2_·HCl, EDAC, TEA, CH_2_Cl_2_, room temperature, 80%; (b) Boc_2_O, 4-(dimethylamino) pyridine, THF, room temperature, 96%; (c) PhSCl, AgOTf, 4 Å molecular sieves, 2,6-di-*tert*-butyl-4-methyl pyridine, CH_2_Cl_2_, 1*R*,2*R*-*trans*-cyclohexanediol in 1:1 THF − CH_2_Cl_2_, −78 °C to room temperature, 13%α-anomer and 60%β-anomer; (d) i. trimethylacetyl chloride, pyridine, triethylammoniun *n*-octadecyl hydrogenphosphonate, room temperature, ii. I_2_, pyridine (9.5 mL) – H_2_O (0.1 mL), room temperature, 58%; (e) i. 0.03 m NaOMe, MeOH, ii. Amberlite IR 120 (H^+^) resin, room temperature, 56%.

The chemical synthesis of the glucocyclitol-phospholipid **11**, Figure 3, began from the carboxylic acid **5** (26). An anomeric mixture of **5** was coupled to *O*-benzylhydroxylamine hydrochloride in the presence of *N*-(3-dimethylaminopropyl)-*N*′-ethylcarbodiimide hydrochloride (EDAC) to afford, after purification, the benzyloxyamide **6** which was treated with di-*tert*-butyl dicarbonate (Boc anhydride) and a catalytic amount of DMAP to give the *N*-Boc-protected hydroxamate **7**. The introduction of the Boc protecting group was necessary in order to circumvent unwanted side reactions, which were apparent in previous studies, when activating the anomeric position for coupling to the cyclohexanediol moiety. The aforementioned coupling was achieved by converting the thioglycoside **7** to a glycosyl triflate through treatment with phenylsulfenyl triflate (PST) (31,32). PST was generated *in situ* by the addition of a freshly prepared solution of 0.4 m benzenesulfenyl chloride (29) to a solution of silver triflate (AgOTf) at −78 °C. After the addition of activated powdered 4 Å molecular sieves, a solution of the thioglycoside **7** and the proton scavenger DTBMP were added at −78 °C, followed by 1*R*,2*R*-*trans*-cyclohexanediol in THF − CH_2_Cl_2_ (1:1). Diligent RBC provided the α- and β-anomers **8** and **9**, at 13% and 60% yields, respectively.

The phosphoric diester **10** was accessible from the pseudodisaccharide **9** by means of the hydrogenphosphonate approach (33). Thus, condensation of the known *n*-octadecyl hydrogenphosphonate salt (27) with the pseudodisaccharide **9** furnished a mixture of diastereoisomeric phosphonic diesters that were converted into the phosphoric diester **10** on oxidation *in situ* with iodine in wet pyridine (33). An identical approach using the α-anomer **8** was unsuccessful owing to inseparable contaminants in the reaction mixture after coupling and *in situ* oxidation.

The generation of the final β-glucohydroxamic acid-cyclitol-phospholipid analog **11** was initially planned to proceed through Zémplen de-*O*-acetylation, followed by the removal of the Boc protecting group under acidic conditions and, finally, catalytic hydrogenolysis to remove the benzyl (Bn)-protecting group. De-*O*-acetylation was carried out using sodium methoxide in methanol and when TLC indicated the complete disappearance of **10**, then the resulting reaction mixture was neutralized with Amberlite IR 120 (H^+^) ion-exchange resin, filtered, evaporated to dryness under reduced pressure, and then subjected to column chromatography. Surprisingly, the major fraction isolated, from the purification, corresponded to the completely deprotected analog **11**: triethylammonium 1*R*,2*R*-*1-O*-[2-*C*-(carboxymethyl *N*-hydroxyamide)-2-deoxy-*β*-d-glucopyranosyl]-cyclohexanediol 2-(*n*-octadecyl phosphate). Thus, de-*O*-acetylation, Boc removal, and benzyl deprotection occurred in a single deprotection step. The ^13^C and ^1^H NMR data indicated the loss of all the protecting groups, which was further confirmed by the mass spectral data. Under the conditions used, de-*O*-acetylation is indisputable, and although it is known from the literature that acid treatment is the common deprotection method used for the Boc group (34), it has also been documented that this group can be removed under basic conditions (35,36). The removal of the benzyl-protecting group of *N*-OBn, on the other hand, is not so obvious. Possibly, after the neutralization of the reaction mixture with Amberlite IR 120 (H^+^) resin followed by its evaporation to dryness, the conditions became sufficiently acidic to lead to the removal of the benzyl-protecting group. This statement is pure conjecture, and the *N*-OBn →*N*-OH reaction pathway may never be understood under the conditions described.

### Evaluation of inhibitors in the trypanosome cell-free system

**Figure 4:**
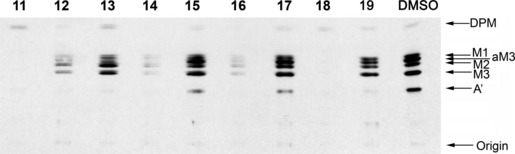
Inhibition of *Trypanosoma brucei* glycosylphosphatidylinositol (GPI) biosynthesis in the cell-free system. Compounds **11**–**19** (10 mm) were incubated with the *T. brucei* cell-free system for 5 min prior to priming with GlcNAc-PI and GDP-[^3^H]Man to stimulate the production of radiolabelled mannosylated GPI intermediates. Glycolipid products were extracted, separated by high-performance thin-layer chromatography, and visualized by fluorography. DPM – dolichol-phosphate-mannose, M1 – Man_1_GlcN-PI, M2 – Man_2_GlcN-PI, M3 – Man_3_GlcN-PI, aM3 – Man_3_GlcN-(acyl)PI, A’– EtN*P*Man_3_GlcN-PI. PI, phosphatidylinositol.

The ability of the compounds **11**–**19** to inhibit the deNAc was initially assessed *in vitro* using the *T. brucei* cell-free system (cfs), i.e., washed trypanosome membranes that are competent in GPI biosynthesis. Because de-*N*-acetylation of GlcNAc-PI must precede the addition of the three mannose residues (13), the activity of the deNAc can be indirectly monitored by measuring production of mannosylated GPI biosynthetic intermediates. In the assay, the cfs is primed with GDP-[^3^H]Man and synthetic GlcNAc-PI **3** with and without inhibitor, and the radiolabelled mannosylated products separated by hptlc, quantified radiometrically, and visualized by fluorography. The compounds **11**–**19** were tested at an initial concentration of 10 mm in the cell-free system (Figure 4). Compounds **13**, **15**, **17,** and **19** produced <10% inhibition of the formation of radiolabelled mannosylated products compared to the DMSO control, while **11**, **12**, **15**, **16,** and **18** all produced >80% inhibition. The potency of the latter compounds was then determined using eight-point potency curves in triplicate (Table 1). Notably, the α/β anomers **17** and **18** gave >100-fold difference in potency. These compounds contain a thiophenyl group, predicted to adopt an axial position in the α-anomer **17,** and an equatorial position in the β-anomer **18**, which could potentially lead to differences in the abilities of the two anomers to fit into the active site of the enzyme. This result is surprising, given that it has previously been observed that the deNAc is able to de-*N*-acetylate both the natural substrate GlcNAc-α-PI **1** and the unnatural GlcNAc-β-PI (17).

**Table 1:** Potency of inhibitors in the indirect cell-free system assay

Compound	IC_50_/μm^a^
**11**	19 ± 3.2
**12**	290 ± 100
**13**	>10 000
**14**	1500 ± 200
**15**	>10 000
**16**	300 ± 50
**17**	>10 000
**18**	100 ± 13
**19**	>10 000

a The IC_50_ values were determined from three separate eight-point potency curves and are reported to two significant figures with standard deviation.

**Figure 5:**
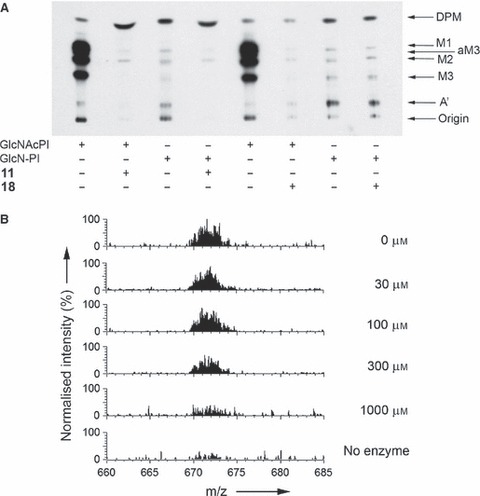
Inhibitors target the GlcNAc-PI de-*N*-acetylase. (A) Inhibition of the *Trypanosoma brucei* cell-free system by **11** and **18** (10 mm) when primed with either GlcNAc-PI or GlcN-PI. Conditions as Figure 4. (B) Inhibition of recombinant *T. brucei* GlcNAc-PI de-*N*-acetylase by **11** measured by electrospray tandem mass spectrometry. The intensity of the reaction product GlcN-I*P*C_18_**4** (*m/z* 672) is normalized to the turnover in uninhibited control. PI, phosphatidylinositol.

The indirect cfs assay is unable to distinguish between the inhibition of the deNAc and inhibition of the first mannosyltransferase (MT1), because either will lead to an overall reduction in mannosylated GPI species. Furthermore, to account for the observation that priming the cfs with GlcNAc-PI is significantly more efficient than priming with GlcN-PI, it has been postulated that substrate channeling occurs between the deNAc and MT1 (14,17). Given the structural similarity of the deNAc substrate GlcNAc-PI **1** and the MT1 substrate GlcN-PI **2**, the analogs **11**–**19** may also interact with MT1. We assessed the specificity of the two most potent inhibitors **11** and **18** by measuring their ability to inhibit the cfs primed with GDP-[^3^H]Man and either the deNAc substrate GlcNAc-PI **1** or the MT1 substrate GlcN-PI **2**. Compound **18** inhibited the formation of mannosylated products when the cfs was primed with GlcNAc-PI **1**, but not when primed with GlcN-PI **2** (Figure 5A), suggesting that it inhibits only the deNAc. Compound **11** inhibited the formation of mannosylated products when the cfs was primed with either GlcNAc-PI **1** or GlcN-PI **2** (Figure 5A), suggesting either that it inhibits both the deNAc and MT1, or that it inhibits MT1 only. The apparent inhibition of MT1 by the glucocyclitol-phospholipid **11** may be due to substrate channeling between the deNAc and MT1, such that inhibition of the deNAc is able to prevent GlcN-PI from accessing the MT1 active site. However, the present data do not rule out direct inhibition of MT1 only.

### Evaluation of inhibitors against recombinant T. brucei GPI de-N-acetylase

We have developed a mass spectrometry-based assay to measure the activity of a recombinant truncated rat deNAc construct, where the first 23 residues corresponding to the transmembrane region are replaced with 6 residues from an orthologous *E. coli* protein (22). Cloning and expression of the equivalent *T. brucei* deNAc construct failed to produce a significant yield of soluble protein. Instead, an alternative construct containing a GST tag (TbGPI12-GST) afforded an improved yield of soluble protein, with protein identity confirmed by tryptic mass fingerprinting (Mascot score 1162, 87% coverage). The activity of TbGPI12-GST was confirmed in the electrospray tandem mass spectrometry (ES-MS/MS) assay using the synthetic GlcNAc-PI analog GlcNAc-I*P*C_18_**3**, where the diacyl glycerol portion of PI is replaced by a C_18_ alkyl chain without affecting enzyme recognition (19). The ability of **11** and **18** to inhibit TbGPI12-GST was assessed in the ES-MS/MS assay, and both were found to inhibit with an IC_50_ = 600 ± 300 μm (Figure 5B) and 980 ± 220 μm (data not shown), respectively. Both compounds show reduced potency against the soluble truncated protein in the ES-MS/MS assay compared to their potency against the intact protein in the indirect cfs assay. This may be due to the loss of interactions with the truncated portion of the protein and/or ER membrane, or in the case of **11**, the lack of the postulated substrate channeling effect.

## Conclusions and Future Directions

Disruption of GPI biosynthesis has been genetically (2–4) and chemically (5) validated as a drug target against *T. brucei*, the causative agent of African sleeping sickness in humans and the related disease Nagana in cattle. African sleeping sickness is invariably fatal if untreated, and there is an urgent need for new therapeutic agents that is not being met by the pharmaceutical industry. We have previously shown that the GPI biosynthetic enzyme GlcNAc-PI de-*N*-acetylase is a zinc metalloenzyme and postulated that ZBGs could act as inhibitors (22). As part of our efforts to develop drugs that target GPI biosynthesis in *T. brucei*, we have synthesized small molecules to probe the mechanism of the GlcNAc-PI de-*N*-acetylase (25,26). Here, we report that small molecules incorporating carboxylic acid or hydroxamic acid can inhibit the *T. brucei* GlcNAc-PI de-*N*-acetylase, confirming our hypothesis that ZBGs can be used to target the enzyme. However, the current compounds are neither sufficiently potent nor drug-like to be useful therapeutics. Future synthetic efforts will be directed toward developing a more drug-like inhibitor backbone, to increase potency, and to introduce species selectivity.

## Abbreviations

deNAc: *N*-acetylglucosamine-phosphatidylinositol de-*N*-acetylase
GPI: glycosylphosphatidylinositol
GlcNAc-PI: *N*-acetylglucosamine-phosphatidylinositol
GlcN-PI: glucosamine phosphatidylinositol
hptlc: high-performance thin-layer chromatography
ZBG: zinc-binding group
